# New Sequence Types and Antimicrobial Drug–Resistant Strains of *Streptococcus suis* in Diseased Pigs, Italy, 2017–2019

**DOI:** 10.3201/eid2801.210816

**Published:** 2022-01

**Authors:** Lucilla Cucco, Marta Paniccià, Francesca Romana Massacci, Alessandra Morelli, Massimo Ancora, Iolanda Mangone, Adriano Di Pasquale, Andrea Luppi, Denis Vio, Cesare Cammà, Chiara Francesca Magistrali

**Affiliations:** Istituto Zooprofilattico Sperimentale dell’Umbria e delle Marche ‘Togo Rosati,’ Perugia, Italy (L. Cucco, M. Paniccià, F.R. Massacci, A. Morelli, C.F. Magistrali);; Istituto Zooprofilattico Sperimentale di Abruzzo e Molise ‘Giuseppe Caporale,’ Teramo, Italy (M. Ancora, I. Mangone, A. Di Pasquale, C. Cammà);; Istituto Zooprofilattico Sperimentale della Lombardia e della Emilia-Romagna ‘Bruno Ubertini,’ Brescia, Italy (A. Luppi);; Istituto Zooprofilattico Sperimentale delle Venezie, Padova, Italy (D. Vio)

**Keywords:** *Streptococcus suis*, infection, serotype, virulence, swine, penicillin, Italy, bacteria, streptococci, antimicrobial resistance

## Abstract

*Streptococcus suis* is a pathogen associated with severe diseases in pigs and humans. Human infections have a zoonotic origin in pigs. To assess circulating strains, we characterized the serotypes, sequence types, and antimicrobial susceptibility of 78 *S. suis* isolates from diseased farmed pigs in Italy during 2017–2019. Almost 60% of infections were caused by serotypes 1/2 and 9. All but 1 of the serotype 2 and 1/2 isolates were confined to a single cluster, and serotype 9 isolates were distributed along the phylogenetic tree. Besides sequence type (ST) 1, the serotype 2 cluster included ST7, which caused severe human infections in China in 1998 and 2005. A large proportion of serotype 9 isolates, assigned to ST123, were resistant to penicillin. The emergence of this clone threatens the successful treatment of *S. suis* infection. Characterizing *S. suis* isolates from pigs will promote earlier detection of emerging clones.

*Streptococcus suis* is a major pathogen in pigs and an emerging zoonotic agent (*1*–*4*). This bacterium is a natural inhabitant of the upper respiratory tract of pigs and is endemic to all pig-production countries. In pigs, *S. suis* causes meningitis, septicemia, polyserositis, arthritis, and endocarditis, mainly during the postweaning period; it is a source of concern for farmers because of potential economic losses and its effects on the welfare of infected pigs (*2*). Human infection is acquired through occupational contact or ingestion of undercooked pork-derived products and is associated with meningitis, endocarditis, septicemia, deafness, and death (*5*).

*S. suis* is a heterogeneous species. Until 2005, *S. suis* was divided into 35 serotypes (1–34 and 1/2), based on capsular polysaccharides, but 6 serotypes were recently reclassified as belonging to other *Streptococcus* species, leaving 29 currently recognized *S. suis* serotypes (*6*,*7*). Most *S. suis* infections in humans and pigs are caused by serotype 2, but the predominant serotypes causing invasive disease in pigs vary according to time and region (*8*). In some countries in Europe, serotype 9 has emerged as the leading cause of invasive diseases in pigs (*2*,*8*–*10*); prevalence of this serotype has also recently increased in China (*5*).

Since 2002, the introduction of a standard multilocus sequence typing (MLST) scheme has improved the description of the epidemiology of *S. suis* infection (*8*). Sequence types (STs), determined by MLST, are also better predictors of the pathogenicity of a particular isolate than are serotypes (*11*). Among serotype 2 isolates from pigs, ST1, a highly successful clone associated with most human infections globally, is prevalent in Europe (*8*). Another sequence type of serotype 2, ST7, was responsible for major *S. suis* epidemics among humans in 1998 and 2005 in China (*12*). Serotypes other than 2 are less frequently responsible for human infections (*8*). Of note, despite the increased frequency of pig infections caused by serotype 9, the first human case of serotype 9 infection was documented in Thailand in 2015 (*13*). That strain was assigned to ST16, an emerging sequence type known for its increased virulence potential and predominance in invasive *S. suis* infections in pigs in the Netherlands (*14*).

On-farm management options for controlling *S. suis* infections include improving environmental conditions (e.g., providing correct temperature, providing correct air humidity, and reducing overcrowding and pig mixing) (*15*). The control of viral infections, particularly porcine reproductive and respiratory virus, is also essential because they are well-known predisposing factors for the disease (*16*). Another tool for protecting against infection is vaccination, but available vaccines are based on bacterins and provide only nonheterologous protection (*17*). Thus, in many countries in Europe, including Italy, control of *S. suis* infections in pigs is based mainly on antimicrobial treatment (*18*). *S. suis* is generally susceptible to β-lactams, the main class of antimicrobials administered to control the infection on pig farms. Conversely, *S. suis* is almost always resistant to tetracycline; macrolide-lincosamide-streptogramin B; and, less frequently, aminoglycosides, chloramphenicol, vancomycin, and linezolid (*15*). In *S. suis*, genes encoding antimicrobial resistance (AMR) are often carried on mobile genetic elements that can be transferred to other members of the genus, including human pathogens (*4*,*19*). Thus, *S. suis* can be considered a public health concern because of its zoonotic potential (a leading cause of antimicrobial drug use in pig farming) and a reservoir of AMR genes (*4*,*19*).

Information about circulating strains is lacking in many countries, including Italy, which is one of the most prominent pig-production countries in Europe (*8*). We characterized the serotypes, sequence types, and antimicrobial susceptibility of 78 *S. suis* isolates from infected pigs in Italy. By providing updated epidemiologic information about *S. suis* infection, we aim to drive the use of autogenous vaccines, reduce antibiotic consumption, and protect animal health. We also assessed presence of *S. suis* clones with zoonotic potential.

## Materials and Methods

### Bacterial Isolates

We investigated isolates collected from pigs with clinical *S. suis* infection on pig farms in northern/central Italy during 2017–2019. To avoid redundancy, we included only 1 isolate per year and farm. A total of 78 *S. suis* isolates were collected from piglets with meningitis (49), pericarditis (1), arthritis (3), septicemia (17), and pneumonia (8) (Appendix 1).

The samples were cultured on 5% sheep blood agar (Biolife Italiana Srl, http://www.biolifeit.com) at 5% CO_2_, 37°C, for 24–48 h. We used matrix-assisted laser desorption/ionization time-of-flight mass spectrometry (Bruker Daltonics GmbH, https://www.bruker.com) and PCR to confirmed selected suspected α-hemolytic colonies as belonging to the *S. suis* species (*20*).

### Serotyping and Virulence Genotyping

We identified serotype and virulence-associated genes by using PCR (Appendix 2 Table 1). To discriminate between different variants of *mrp*, we used whole-genome sequencing (*21*–*23*).

### Antimicrobial Susceptibility Testing

We assessed MICs by using a commercially prepared microtiter MIC panel (BOP06F, Sensititre; Trek Diagnostic Systems Inc., https://www.thermofisher.com) according to the manufacturer’s instructions and by using *Streptococcus pneumoniae* ATCC 49619 as a quality control strain. We interpreted MIC results by using the breakpoints recommended by the Clinical Laboratory Standards Institute (*24*) for swine respiratory *S. suis*. The interpretative criteria for trimethoprim/sulfamethoxazole and clindamycin were those recommended for human *S. pneumoniae* (*25*).

### Whole-Genome Sequencing

We prepared genomic DNA from all 78 *S. suis* isolates. We extracted pure cultures from 1 mL of logarithmic-phase broth cultures by using QIAamp DNA Mini Kit (QIAGEN, https://www.qiagen.com) according to the manufacturer’s instructions and then quantified the DNA by using the Qubit fluorometer (Thermo Fisher Scientific, https://www.thermofisher.com). We prepared the libraries by using the Nextera XT Library Prep kit (Illumina Inc., https://www.illumina.com) and then loaded them onto an Illumina NextSeq 500/550 Mid Output Reagent Cartridge version 2 kit (300 cycles) and sequenced them on an Illumina NextSeq 500 platform to generate 150-bp paired-end reads.

### Sequence Analyses

Raw data were checked for quality, trimmed by using Trimmomatic version 0.36 (*26*), and assembled by using SPAdes genome assembler version 3.11.1 (*27*). To determine distinct sequence types, we performed MLST. The allele sequences and profiles were obtained from the *S. suis* MLST database (https://pubmlst.org/ssuis). We uploaded sequences for new MLST allele variations to the same database for assignment of allele identification and then uploaded final allele combinations for assignment of new MLSTs. We submitted the raw sequencing data to the National Center for Biotechnology Information Sequence Read Archive repository (BioProject PRJNA717238, Biosample SUB9357225; accession nos. SAMN18490763–SAMN18490790).

To identify potential clonal complexes and founders, we performed global optimal eBURST (http://www.phyloviz.net/goeburst analysis). The entire *S. suis* MLST database was displayed as a single goeBURST diagram by setting the double-locus variants level and the group definition to 0 of 7 shared alleles. We conducted minimum core-genome sequence typing in silico (*28*).

We annotated genomes by using Prokka (https://github.com) and constructed a maximum-likelihood phylogenetic tree, based on the final alignment of core genome from Roary analysis, by using FastTree 2.1.11 (*29*). Manual annotation of the tree was performed in iTOL (v.5.7) (*30*). We identified AMR genes by using ABRicate (https://github.com) against the following databases: AMRFinderPlus, CARD, RESfinder, ARG-ANNOT (*31*–*34*).

To research putative virulence genes, we created a database containing 91 previously described genes (*2*,*3*) (Appendix 2 Table 1) and searched by using BLASTN version 2.5.0+ (*35*). According to O’Dea et al., (*17*), only genes with >95% coverage and >99% identity were considered present. We investigated the null hypothesis of a random distribution of the number of virulence factors among the different sequence types and excluded sequence types represented by a small set of isolates (<3), resulting in 8 sequence types and 65 isolates. To show the distribution of the putative virulence genes across the sequence types, we selected the putative virulence genes that were present in <90% or in >10% of isolates. After checking the normality of the data by using the Shapiro-Wilk normality test, we performed Kruskal-Wallis rank-sum testing, followed by pairwise comparisons using the Dunn test for multiple comparisons of independent samples. To investigate the distribution of genes encoding putative virulence factors, we constructed a heat map based on the distance metric “euclidean” and complete linkage method. We performed all analyses in R (*36*).

## Results

### Molecular Serotyping, Virulence Genotyping, and MLST

We identified 13 serotypes: 1, 2, 1/2, 3, 4, 5, 7, 8, 9, 10, 15, 19, and 23. The most prevalent were serotypes 9, accounting for 34.6% (n = 27) of isolates, and 1/2, accounting for 25.6% (n = 20) of isolates. These serotypes were followed by 10 (n = 7, 9.0%), 2 (n = 7, 9.0%), and 7 (n = 6, 7.7%) (Table 1).

**Table 1:** Combination of putative virulence genes among sequence types and minimum core genome groups of *Streptococcus suis* from diseased pigs, Italy, 2017–2019*

Sequence type	MCG group	Serotype	Virulence profile	No. isolates/total no. isolates for each sequence type (%)
ST1	1	2	*mrp^EU^/sly/epf*	3/17 (17.6)
ST1	1	1/2	*mrp^EU^/sly/epf*	14/17 (82.4)
ST7	1	1/2	*mrp^EU^ /sly/epf*	6/9 (66.6)
ST7	1	2	*mrp^EU^ /sly/epf*	3/9 (33.3)
ST11	N	1	*mrp/sly/epf*	2/2 (100)
ST16	1	9	mrp*/sly	3/3 (100)
ST28	4	2	*mrp^NA1^/sly*	1/1 (100)
ST29	4	7	mrp*	3/6 (50)
			*mrp^NA1^*	3/6 (50)
ST94	3	4	*mrp^NA1^/sly*	2/3 (66.7)
		9	*mrp^NA1^/sly*	1/3 (33.3)
ST108	3	23	*mrp^NA1^/sly*	1/1 (100)
ST123	3	9	*mrp^NA1^/sly*	17/17 (100)
ST1540	N	9	-	3/3 (100)
ST1541	1	9	-	1/1 (100)
ST1542	N	3	-	1/1 (100)
ST1543	3	4	*mrp^NA1^/sly*	1/1 (100)
ST1544	3	4	*mrp^NA1^/sly*	1/2 (50)
		5	*mrp^NA1^/sly*	1/2 (50)
ST1545	1	8	mrp*	1/1 (100)
ST1546	1	8	*mrp/sly*	1/1 (100)
ST1547	1	10	*-*	7/7 (100)
ST1548	N	15	*sly*	1/1 (100)
ST1549	N	19	*-*	1/1 (100)

*Dashes indicate absence of putative virulence genes according to PCR. mrp* is the *mrp* variant (*22*). MCG, minimum core genome; N, not groupable; ST, sequence type.

MLST analysis revealed that 59 (75.6%) isolates belonged to 9 sequence types (ST1, ST7, ST11, ST16, ST28, ST29, ST94, ST108, and ST123) in the *S. suis* MLST database, and 10 new sequence types were identified as ST1540–1549 (ID2702-ID2711; https://pubmlst.org/organisms/streptococcus-suis). Most were singletons, except for ST1547 (n = 7, all serotype 10), ST1540 (n = 3, serotype 9), and ST1544 (n = 2, serotypes 4 and 5). With 17 isolates each, the predominant STs were ST1 and ST123, which accounted for 43% of all isolates. All isolates belonging to ST29 were serotype 7, and all isolates belonging to ST11 were serotype 1.

### AMR Phenotypes and Genotypes

**Figure 1:**
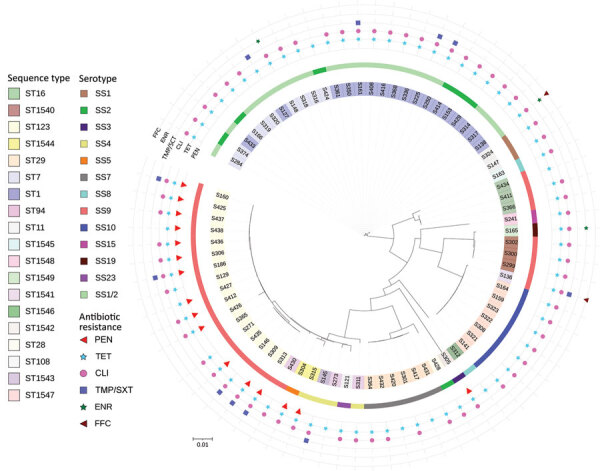
Circular phylogenetic tree containing 78 *Streptococcus suis* isolates from diseased pigs, Italy, 2017–2019. The tree was inferred by using the iTOL interactive user interface (https://itol.embl.de). Shading over tip labels indicates sequence types. The serotypes of each isolate are also shown. The antimicrobial-resistant molecules are annotated by colors and shapes. Scale bar indicates substitutions per site. CLI, clindamycin; ENR, enrofloxacin; FFC, florfenicol; PEN, penicillin; ST, sequence type; TET, tetracycline; TMP/SXT, trimethoprim/sulfamethoxazole.

A total of 7 (9.0%) *S. suis* isolates were resistant to antimicrobial drugs, usually tetracycline (6/7) (Table 2). Most (48/78, 61.5%) isolates were resistant to 2 antimicrobials, generally (45/48) clindamycin and tetracycline. Of the 78 isolates, 23 (29.5%) were resistant to >3 antimicrobials and were classified as multiresistant. Multiresistance was detected in 4/17 (23%) ST1, 1/9 (11%) ST7, and 12/17 (71%) ST123 isolates. Of 17 ST123 isolates, 14 (82%) were resistant to penicillin (Tables 1, 3; Figure 1).

**Table 2:** Distribution of MICs among 78 *Streptococcus suis* isolates from diseased pigs, Italy, 2017–2019*

Antibiotic molecule	No. (%) isolates by MIC, μg/mL												
	0.12	0.25	0.5	1	2	4	8	16	32	64	128	256	512
Penicillin G	49 (63)	7 (9)	5 (6)	14 (18)	3 (4)								
Ampicillin		73 (94)	2 (3)	3 (4)									
Enrofloxacin		1 (1)	17 (22)	57 (73)	2 (3)	1 (1)							
Tetracycline				1 (1)	3 (4)	1 (1)		73 (94)					
Florfenicol		1 (1)	3 (4)	40 (51)	32 (41)		2 (3)						
TMP/SXT					67 (86)	11 (14)							
Clindamycin†		8 (10)				4 (5)	4 (5)	10 (13)	50 (64)				
Tylosin†			15 (19)							61 (78)			
Neomicin†						22 (28)	27 (35)	15 (19)	7 (9)	5 (6)			
Gentamicin†				12 (15)	39 (50)	17 (22)	6 (8)		2 (3)				
Sulfadimethoxine†											19 (24)		57 (73)

*Gray shading indicates range of values actually tested for each antibiotic. Black vertical bars indicate threshold values for clinical resistance, according to the Clinical and Laboratory Standards Institute (https://clsi.org). TMP/SXT, trimethoprim/sulfamethoxazole. †Two isolates were not identified.

**Table 3:** Antimicrobial resistance genes identified in the 78 *Streptococcus suis* isolates from diseased pigs, Italy, 2017–2019, by sequence type

Antimicrobial resistance genes	Sequence type									Total
	ST1	ST123	ST1547	ST29	ST7	ST16	ST94	ST1540	Other*	
*ermb*, *tet(O)*	11	15	3	3	8	3	1		2	46
*tet(O)*	1		4		1					6
None				1			1		3	5
*Cv ermb*, *tet(O)*, *dfr(F)*	5									5
*aac6-aph2*, *ant6-ia*, *aph3-iiia*, *spw*, *ermb*, *tet(**40**)*, *tet(W)*, *tet(O)*, *tet(O/W/32/O)*, *tet (W/N/N)*								3	1	4
*tet(M)*				1						1
*ermb*, *tet(M)*				1						1
*ermb*, *tet(W)*, *tet (O/W/32/O)*, *tet(W/N/N)*									1	1
*ant6ia*,*aadE*, *ermb*, *tet(O)*									1	1
*aac6-aph2*		1								1
*ant6ia*, *aph3-iiia*, *spw*, *cat*							1			1
*ant6ia*, *aph3-iiia*, *apmA*, *ermb. optrA*, *tet(**40**)*, *spw*									1	1
*aac6-aph2*, *ermb*, *tet(O)*									1	1
*ant6-ia*, *aadE*, *ermb*, *tet(W)*, *tet(O)*, *tet(O/W/32/O)*, *tet(W/N/N)*									1	1
*ant6-ia*,*aadE*, *ermb*, *tet(**40**)*, *tet(O)*, *tet(O/32/O)*									1	1
*ant6-ia*, *spw*, *lnuB*, *lsaE*, *tet(O)*									1	1
*aac-aph2*, *aad(**6**)*, *spw*, *ermb*, *erm(**47**)*, *lnuB*, *lsaE*, *tet(**40**)*, *tet(T)*		1								1
Total	17	17	7	6	9	3	3	3	13	78

*Sequence types (STs) represented by <3 isolates.

### Phylogenetic Analyses

A total of 1,156 genes, corresponding to 19.88% of the pangenome (Appendix 2), comprised the core genome. Phylogenetic analysis of the collected isolates showed 4 major clusters and 2 singletons. The first cluster was composed of serotypes 1, 1/2, and 2 isolates and was characterized by low heterogeneity, even though these isolates originated from different regions and body sites. We found no relationship among the 4 isolates of the second cluster, belonging to serotypes 8 and 9. Cluster 3 recognized 2 sister groups: the first group comprised serotypes 9, 15, and 19; the second group included serotype 10 isolates. The serotype SS10 isolates were derived from 7 outbreaks of meningitis, 6 of which occurred in the Piedmont region of northern Italy in 2018. The fourth cluster included the highest number of isolates from our collection, belonging to 6 serotypes. We found no correlations with geographic location, year, or site of origin for members of this cluster. All penicillin-resistant SS9 isolates were grouped within this cluster (Figure 1). goeBurst analysis (http://www.phyloviz.net/goeburst) showed 5 major clusters. ST1543 and ST1544 were related to the ST94 subgroup, ST1546 to the ST1521 subgroup, and ST1545 to clonal complex (CC) 1; the other sequence types occurred as singletons (Appendix 2 Figure 1).

### Putative Virulence Genes

**Figure 2:**
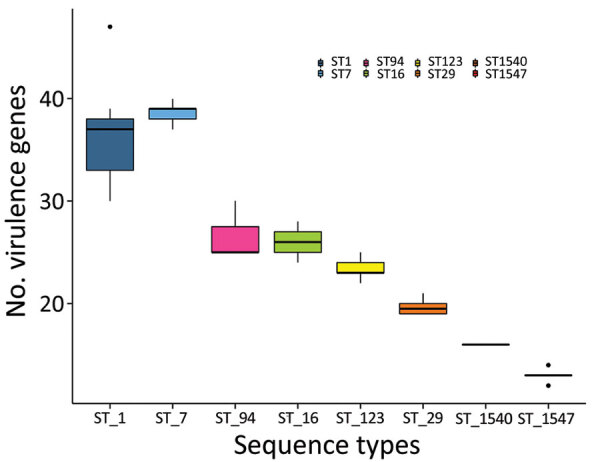
Distribution of the putative virulence genes detected among different sequence types of *Streptococcus suis* isolates from diseased pigs, Italy, 2017–2019. Box tops and bottoms indicate interquartile ranges, horizontal lines within boxes indicate means, whiskers indicate 95% CIs, and dots indicate outliers. ST, sequence type.

When we investigated the distribution of putative virulence genes in a subset of 65 isolates belonging to 8 sequence types (ST1, ST7, ST16, ST29, ST94, ST123, ST1540, and ST1547), we found 61 putative virulence genes in >10% and <90% of the isolates and included them in the heat map. Putative virulence genes were not randomly distributed across the 8 sequence types (p<0.001 by Kruskal-Wallis test; Figure 2). The number of putative virulence genes in ST1 and ST7 isolates did not differ. The number of putative virulence genes in ST1 and ST7 isolates differed from the number in ST123, ST29, ST1540, and ST1547 (p<0.05 by Dunn test). A block of 38 putative virulence genes was characteristic of ST1 and ST7 isolates. This block included genes encoding components of the cell wall, proteases, and molecules related to adhesion (*cps2E*, *cps2F*, *cps2C*, *neuB*, *fbps*, *sbp2*, *pgdA*, *dppIV*, *igaP*, *ssnA*, *srtF*, and *gnd*) and putative virulence genes involved in the regulation of metabolic pathways (*ccpA*, *lspA*, *ssu1889*, *revS*, *virA*, *guaB*, *sodA*, *adcR*, *purA*, *nadR*, *stp*, *stk*, *vapE*, *lysS*, *Ssads*, *fhs*, *apuA*, *aroK*, *flps*, *proA*, *scrB*, *ofs*, *prtP*, *gtfA*, *perR*, and *fur).* Virulence genes harbored by the 89-kb pathogenicity island, including *SalK/SalR* and *tetM*, were not found in ST7 isolates. ST123 was characterized by the presence of another group of 12 putative virulence genes related to adhesion (*murM*) or involved in metabolic pathways (*htpsC*, *ppc*, *troA*, *pyrF*, *nox*, *purD*, *msmK*, *gloA*, *rgg*, and *yhbU_2*, *lysM*) (Appendix 2 Figure 3).

## Discussion

Among 78 *S. suis* isolates from diseased pigs in Italy, we identified the major serotypes associated with *S. suis* infections as serotypes 9 and SS1/2, responsible for almost 60% of cases. Previous studies in Italy have shown a predominance of serotype 2 infections, which were a minority in our study (*1*,*37*). Until 2020, PCR testing for serotyping did not differentiate serotype 2 from serotype 1/2, and many studies reported these 2 serotypes as belonging to the same category. However, such distinction is relevant because serotype 1/2 is associated with pig infections; however, different from serotype 2, its role as a zoonotic agent is still uncertain (*38*). With this study, we confirmed increased serotype 9 infections at pig farms, which has already been described for other countries in Europe (*10*). The proportion of isolates belonging to serotype 7, the third most common serotype, remained more or less stable compared with data from 2000, thus confirming the trend observed in Germany (*7*,*37*). We also detected serotypes 10 and 15 in our collection. These serotypes have not previously been detected in Italy but have been identified in Spain and the United Kingdom (*37*). Given the variability of serotypes and the low prevalence of serotype 2 observed in our study, complete characterization of isolates is essential for the successful implementation of autogenous vaccines. Indeed, autogenous vaccines are well-established tools for preventing serotype 2 infections, but data about their efficacy for other serotypes, including 1/2, are still lacking (*16*).

All serotype 2 and 1/2 isolates, except 1, were confined to a single cluster of the phylogenetic tree. This cluster was composed of ST1 and, unexpectedly, ST7, which is a subgroup founder related to CC1. The number of putative virulence factors was higher in ST7 and ST1 isolates than in other STs in our collection, which was expected, because ST1 is the predominant sequence type associated with invasive infections in pigs in Europe. Cases of *S. suis* infection in humans in Italy are sporadic and are caused by serotype 2, ST1 (*1*,*39*). ST7, which differs from ST1 at a single locus, has not been detected in pigs in Europe (*8*). However, ST7 isolates are prevalent among diseased pigs in China (*40*–*42*). The epidemic strain ST7, which is characterized by the presence of an 89-kb pathogenicity island, the insertion of a 128-kb ICE (integrative and conjugative element)–phage tandem mobile genetic element, is responsible for the 2 largest outbreaks of human *S. suis* infection, which occurred in 1998 and 2005 (*42*). The ST7 isolates from our study lacked the virulence genes harbored by the 89-kb pathogenicity island; thus, their zoonotic potential may be lower than that of the ST7 epidemic strain in China. Moreover, they did not cluster with the newly described lineage III of ST7 (Appendix 2 Tables 2–4, Figure 2) (*43*). Further analysis is necessary to explain the presence of ST7 in Italy. New *S. suis* strains may be imported by living animals or traveling humans, or they may have been derived from an early mixing of pig breeds, as previously hypothesized (*3*,*40*).

All serotype 7 isolates belonged to ST29, grouped in cluster 4, and had 2 *mrp* gene variants. The same characteristics were described for serotype 7 ST29 isolates from recent severe outbreaks among piglets in Germany and Austria (*18*). Thus, ST29 has been suggested as an emerging virulent sequence type in Europe (*18*).

In contrast to the serotype 2 and 1/2 isolates, serotype 9 isolates were distributed among different clusters in the phylogenetic tree, grouping with isolates belonging to other serotypes. High heterogeneity has been reported for serotype 9 (*11*,*44*). Three isolates from our collection were typed as serotype 9 ST16, a dominant clone in diseased pigs from the Netherlands (*3*). Although most cases in humans have been attributed to ST1 isolates, ST16 has recently been associated with cases of *S. suis* infection in humans in Thailand (*13*). It has been suggested that the zoonotic and virulence potential may be higher for ST16 than for other strains. In our study, the ST16 subgroup was related to CC1, harbored *mrp* and *sly* genes, and was close to ST1 and ST7 in the phylogenetic tree, in accordance with the results reported by Zheng et al. (*10*). The presence of ST16 in Italy suggests the need for monitoring and typing *S. suis* from diseased pigs and infected humans in a One Health scenario.

A large proportion of serotype 9 isolates were assigned to ST123 and grouped into cluster 4 in the phylogenetic tree. This sequence type was prevalent in our collection and was found in 5 regions of Italy and in pigs from different production companies. Most ST123 isolates were resistant to penicillin. ST123 was reported in Spain in 2009 (*9*). As already observed in Spain, the ST123 isolates from our study were related to the ST94 subgroup and were characterized by the presence of *sly* and *mrp^NA1^* genes (*10*).

The number of live pigs imported into Italy has increased over the past 10 years, almost doubling from 2013 to 2018 (http://www.anas.it). Pigs are imported from other countries in Europe, predominantly the Netherlands, Denmark, and Germany, and, to a lesser extent, from Spain and countries in eastern Europe. Imported live pigs can be carriers of new *S. suis* clones, which may then be transferred to other animals at the receiving farm (*11*). The differences in the *S. suis* population in our study compared with those in previous studies may result from this intensive exchange of live pigs between Italy and other countries in Europe.

We confirmed widespread resistance to tetracycline and clindamycin, as previously reported for *S. suis* isolates globally. Resistance to tetracycline was mainly associated with the presence of *tetO* and, to a lesser extent, other *tet* genes, including *tetM* and the mosaic gene *tet (O/W/32/O)*, which was first described in *S. suis* isolates in Italy (*1*). Resistance to clindamycin was coupled with high MICs for tylosin and the diffuse presence of *ermB*, suggesting a macrolide/lincosamide/streptogramin B profile. Resistance to florfenicol was detected in 2 multiresistant isolates. This type of resistance is emerging in *S. suis* species (*45*). One of the florfenicol-resistant isolates was positive for *optrA*, an oxazolidinone/phenicol resistance determinant carried by mobile genetic elements. *optrA* in *S. suis* isolates from China has been previously described and is frequently detected in *Enterococcus* isolates from pig farms in Italy (*46*,*47*). The high levels of AMR and the detection of emerging drug-resistance determinants are a consequence of selective pressure caused by antibiotic overuse. Despite the declining trend in antibiotic consumption, the use of antibiotics in veterinary medicine is still more frequent in Italy than in other countries in Europe (*48*).

We observed a high level of resistance to penicillin; ≈1 in 5 isolates showed reduced susceptibility to this antimicrobial. This finding contrasts with previous observations from other countries in Europe (*15*,*49*). Resistance to ampicillin was not observed, thus confirming the hypothesis of incomplete cross-resistance between these 2 antimicrobials (*49*). Resistance to penicillin was mostly detected in serotype 9 isolates and was particularly frequent in ST123 isolates. Blume et al. (*9*) suggested that the spread of *S. suis* serotype 9 is favored by the selective advantage conferred by the absence of heterologous immunity induced by the dominant serotype 2 clone (*9*). Our data suggest that penicillin resistance may also be a driver of the expansion of *S. suis* serotype 9.

The emergence of a penicillin-resistant clone among the *S. suis* population threatens the successful treatment of *S. suis* infections in pigs. Penicillin resistance in *S. suis* may favor the prescription of critical classes of antimicrobial drugs, which should be limited in veterinary medicine. Penicillin resistance in a zoonotic agent raises concerns about hampering the treatment of infections.

In conclusion, our study highlights the value of characterizing *S. suis* isolates from pigs for monitoring trends in AMR and enabling early detection of emerging clones. In addition, our data strongly suggest the need for preventive strategies to limit the spread of penicillin-resistant *S. suis* among pig populations in Italy.

Appendix 1 Complete dataset for isolates from pigs with clinical *Streptococcus suis* infection, northern/central Italy, 2017–2019.

Appendix 2 Supplemental results for study of new sequence types and antibiotic-resistant strains of *Streptococcus suis* in diseased pigs, Italy, 2017–2019.
