# Supplementary material for: New Sequence Types and Antimicrobial Drug–Resistant Strains of Streptococcus suis in Diseased Pigs, Italy, 2017–2019
Source: Emerg Infect Dis. 2022 Jan;28(1):139–47. doi: 10.3201/eid2801.210816 (PMC8714200; doi:10.3201/eid2801.210816)
Supplement: Appendix 2 — Supplemental results for study of new sequence types and antibiotic-resistant strains of Streptococcus suis in diseased pigs, Italy, 2017–2019. [file 21-0816-Techapp-s2.pdf]

Article DOI: https://doi.org/10.3201/eid2801.210816

# New Sequence Types and Antimicrobial Drug–Resistant Strains of *Streptococcus suis* in Diseased Pigs, Italy, 2017–2019

## Appendix 2

### Read quality, number of contigs and coverage of Whole-Genome Sequencing data

The final assembly of the 78 sequences resulted in an average read quality after trimming of 34.60% (min 34.04, max 34.89) and 3,165,852 read pairs (min 1,043,985; max 5,077,416). The average number of contigs was 68 (min 25, max 177), with a mean length of 2,114,359 nucleotides (min 2,026,262; max 2,264,709). The average vertical coverage was 211 (min, 69; max, 339). The mean N50 and L50 values were 149,502 nucleotides (min 371, 66; max 293,044) and 6.95 (min 3, max 21), respectively.

#### References

1. Okura M, Lachance C, Osaki M, Sekizaki T, Maruyama F, Nozawa T, et al. Development of a two-step multiplex PCR assay for typing of capsular polysaccharide synthesis gene clusters of Streptococcus suis. J Clin Microbiol. 2014;52:1714–9. PubMed https://doi.org/10.1128/JCM.03411-13

2. Lacouture S, Okura M, Takamatsu D, Corsaut L, Gottschalk M. Development of a mismatch amplification mutation assay to correctly serotype isolates of *Streptococcus suis* serotypes 1, 2, 1/2, and 14. J Vet Diagn Invest. 2020;32:490–4. PubMed https://doi.org/10.1177/1040638720915869

3. Silva LMG, Baums CG, Rehm T, Wisselink HJ, Goethe R, Valentin-Weigand P. Virulence-associated gene profiling of Streptococcus suis isolates by PCR. Vet Microbiol. 2006;115:117–27. PubMed https://doi.org/10.1016/j.vetmic.2005.12.013

4. Wisselink HJ, Reek FH, Vecht U, Stockhofe-Zurwieden N, Smits MAE, Smith HE. Detection of virulent strains of Streptococcus suis type 2 and highly virulent strains of Streptococcus suis type 1 in tonsillar specimens of pigs by PCR. Vet Microbiol. 1999;67:143–57. PubMed https://doi.org/10.1016/S0378-1135(99)00036-X

5. King SJ, Heath PJ, Luque I, Tarradas C, Dowson CG, Whatmore AM. Distribution and genetic diversity of suilysin in Streptococcus suis isolated from different diseases of pigs and characterization of the genetic basis of suilysin absence. Infect Immun. 2001;69:7572–82. PubMed https://doi.org/10.1128/IAI.69.12.7572-7582.2001

6. Princivalli MS, Palmieri C, Magi G, Vignaroli C, Manzin A, Camporese A, et al. Genetic diversity of Streptococcus suis clinical isolates from pigs and humans in Italy (2003-2007). Euro Surveill. 2009;14:19310. PubMed https://doi.org/10.2807/ese.14.33.19310-en

7. Dong X, Chao Y, Zhou Y, Zhou R, Zhang W, Fischetti VA, et al. The global emergence of a novel Streptococcus suis clade associated with human infections. EMBO Mol Med. 2021;13:e13810. PubMed https://doi.org/10.15252/emmm.202013810

**Appendix 2 Table 1.** Primers used to identify the *S. suis* serotypes and virulence factors by PCR*

| Target | Primer sequences (5′ to 3′) Forward | Reverse | Reference |
|---|---|---|---|
| For grouping PCR | | | |
| I | TGGTTCAAATATCAATGCTC | ATTGGTTGTGAGTGCATTG | (*1*) |
| II | TCAAAATACGCACCTAAGGC | CACTCACCTGCCCCAAGAC | |
| III | TGATTTGGGTGAGACCATG | CTCATGCTGGATAACACGT | |
| IV | ACAGTCGGTCAAGATAATCG | TCAGCTTGGGTAATATCTGG | |
| V | GGAAAGATGGAGGACCAGC | CCAACCAGACTCATATCCCC | |
| III and VI | GATGCCCCAAGCGATATGCC (F1) | GGACCAACAATGGCCATCTC (R1) | |
| | GACGCACCAAGTGATATGCC (F2) | GGTCCGACAATAGCCATTTC (R2) | |
| For typing PCR | | | |
| SS group I | | | (*1*) |
| 3 | GGTTTTGATTGGTCTAGTTG | CTCTAAAGCTCGATATCTAC | |
| 13 | TATGGTTAAAGGTGGAACTG | CCTTGTATATATTCCCTCCA | |
| 18 | TAATGGGATAGTTGCGTTAC | ATACATAAAGTTGTCCTGCG | |
| SS group II | | | (*1*) |
| 2 | TTAGCAACGTTGCCAATAAG | AATCCTCCATTAAAACCCTG | |
| 6 | GCTCACTATTTTTACATTACAC | TATTACTCCGCCAAATACAG | |
| 1 and 14 | TTAGACAGACACCTTATAGG | CTAGCTTCGTTACTTGATTC | |
| 16 | AAGGTTATCCACGAAAGATG | TCCGGCAATATTCTTTCAAG | |
| 27 | AGACACTGCTTGCATTATTG | TCAGAATTACTTCCTGTTGC | |
| 1 and 1/2 | ATCGCTTTGTGGTGGCCTTT | AGAAGCTTCTTTTGCTGTTTGC | (*2*) |
| 1 and 14 | ATCGCTTTGTGGTGGCCTTG | | |
| SS group III | | | (*1*) |
| 21 | TATCATATTGAGAATCTTCCC | TTGCGTAGCATACAAAGTTC | |
| 28 | ATTATGTTGGTTGCAGAAGG | CGACTCAATTGTTGTAGTAG | |
| 29 | TTCTGGGATTTTAGGAATGC | CATGAAATACGCACTTGTAC | |
| 30 | TATTGCACTAGCTTCAGAAC | TGCATCCATAGTTGTATTCG | |
| SS group IV | | | (*1*) |
| 4 | GACTATCTGTATACCCAAAC | TCCTTCCAAGTATTCTCTAG | |
| 5 | ATCTTAGGAATGATTCGGAC | ACCAGATATCTGAGCAAATG | |
| 7 | AACTACCTACCTGAACTTTG | AGTCTAAAAGTGATCGAGTC | |
| 17 | TAGCATCAGTTTATACGAGG | TAGTTTATCTGTGACACACC | |
| 19 | GTGTCGCAAATCAAGTATTG | AAGCTAGTACAACAAGCATG | |
| 23 | TAATGTATGCTCTGTCACTG | AACGAAACGGAATAGTTTGC | |
| SS group V | | | (*1*) |
| 8 | AAATAAGGTAGGAGCTACTC | ATCCAACCTTAGCTTTCTGT | |
| 15 | ATCGTTTTGAGATTGAGTGG | TAAACGGATTCGGTTACTCA | |
| 20 | TGTGGATTTCTGGGATAATC | TGTGGACGAATTACTACTTG | |
| 22 | GCATTATCAGGATTCTTTCC | CCAATTGGGTGTTCAAAAAG | |
| 25 | GTTTGCTCCGATCATAATAG | CCAGTAAAAGGACTCAATAC | |
| SS group VI | | | (*1*) |
| 9 | GAAAGTAGGTATATCTCAGC | GGGCTATTAAAACTCCTATC | |
| 10 | TTTCCCATTTGCTTATGGAC | GGAATAAAAACGATTGGGAG | |
| 11 | ATGCGATTGCAACAATTGAC | AGGCATGAGTAATACATAGG | |
| 12 | AACAGGTATTTCAGGATTGC | CTCGGATAAAGATAATCAGC | |
| 24 | TACTGAGATTTATTGGGACG | AAGCGATTGGATTACATTGC | |
| 26 | TTATACCGAAATTTTGTTGCC | CGTCAATCATATAAAGTGGG | |
| 33 | GATGTTTTCAACAGGTGTAC | CAAAGTACCTATTTTCAGCG | |
| SS group VII | | | (*1*) |
| 31 | ACAATCGTTTCTGCAATACG | GATGAAAACATCGTTGGTAG | |
| | ATCAGTAGTGGGAATAGTTG | TTTACTGTTTTTCGACCGTG | |
| 32 | AACCGCTGTTGAATTAAGAG | TTCGTTAGTTGAACTGTTCC | |
| | TAGGACTATGGTTCCTAATG | TATTCTAGTTCAAGTCGCTC | |
| 34 | AAGTTTCATTCGAGGACTTC | GTATATAACACCGCAAGAAG | |
| | ATACAGTGATGTCTTGCAAC | ATTGCTTTTTGACAATCGGC | |
| For virulence factors | | | |
| mrp | GACAGATGGTGAGGAAAATGG | TGAGCTTTACCTGAAGCGGT | (*3*) |
| epf | GCTACGACGGCCTCAGAAATC | TGGATCAACCACTGGTGTTAC | (*4*) |
| Sly | CAGCTCGTTGCCTTGTACTA | ACTCTATCACCTCATCCGC | (*5*) |

*The DNA for SS3 and SS14 were kindly provided by Marcelo Gottschalk (University of Montreal, Canada) and used as positive control. *S. suis* strain V20 from a previous study conducted in Italy (*6*) was used as control.

**Appendix 2 Table 2.** Genes lacking in Lineage III and Lineage I ST7 reference strains, but present in the ST7 isolates of this study

| Gene | Annotation |
|---|---|
| group_16 | putative autolysin SsaALP |
| group_2145 | IS630 family transposase ISSsu3 |
| elaA | Protein ElaA |
| group_324 | hypothetical protein |
| group_325 | hypothetical protein |
| group_326 | hypothetical protein |
| group_327 | hypothetical protein |
| pdxK | Pyridoxine kinase |
| hmpT | Thiamine precursor transporter HmpT |
| group_330 | hypothetical protein |
| group_331 | hypothetical protein |
| baeS | Signal transduction histidine-protein kinase BaeS |
| walR_2 | Transcriptional regulatory protein WalR |
| group_334 | hypothetical protein |
| mco | Multicopper oxidase mco |
| group_336 | IS3 family transposase ISStin7 |
| group_337 | hypothetical protein |
| group_338 | hypothetical protein |
| group_339 | hypothetical protein |
| group_340 | hypothetical protein |
| immR | HTH-type transcriptional regulator ImmR |
| mhqD | Putative hydrolase MhqD |
| mhqA | Putative ring-cleaving dioxygenase MhqA |
| pgl_1 | 6-phosphogluconolactonase |
| group_347 | hypothetical protein |
| lgt_1 | Phosphatidylglycerol--prolipoprotein diacylglyceryl transferase |
| group_349 | hypothetical protein |
| group_350 | hypothetical protein |
| copB | Copper-exporting P-type ATPase B |
| group_352 | hypothetical protein |
| actP | Copper-transporting P-type ATPase |
| copY_2 | Transcriptional repressor CopY |
| cadC_1 | Cadmium resistance transcriptional regulatory protein CadC |
| group_356 | hypothetical protein |
| group_357 | hypothetical protein |
| group_358 | hypothetical protein |
| group_359 | hypothetical protein |
| group_360 | hypothetical protein |
| cadC_2 | Cadmium resistance transcriptional regulatory protein CadC |
| cadA | putative cadmium-transporting ATPase |
| yadH | Inner membrane transport permease YadH |
| yadG | putative ABC transporter ATP-binding protein YadG |
| cdr_1 | Coenzyme A disulfide reductase |
| acr3 | Arsenical-resistance protein Acr3 |
| arsA | Arsenical pump-driving ATPase |
| group_368 | hypothetical protein |
| arsD | Arsenical resistance operon trans-acting repressor ArsD |
| group_37 | Deoxyguanosinetriphosphate triphosphohydrolase-like protein |
| group_370 | IS6 family transposase IS1216V |
| group_371 | hypothetical protein |
| group_372 | hypothetical protein |
| dnaG_1 | DNA primase |
| group_374 | hypothetical protein |
| group_375 | hypothetical protein |
| group_376 | hypothetical protein |
| group_377 | hypothetical protein |
| ltrA | Group II intron-encoded protein LtrA |
| group_379 | hypothetical protein |
| group_380 | hypothetical protein |
| group_381 | hypothetical protein |
| group_382 | hypothetical protein |
| group_383 | hypothetical protein |
| group_45 | hypothetical protein |
| yeeO_3 | putative FMN/FAD exporter YeeO |
| group_82 | hypothetical protein |
| sdpR_1 | Transcriptional repressor SdpR |

**Appendix 2 Table 3.** Genes lacking in in the ST7 isolates of this study, but present in Lineage I ST7 reference strains

| Gene | Annotation |
|---|---|
| group_310 | Tyrosine recombinase XerC |
| group_311 | hypothetical protein |
| group_312 | hypothetical protein |
| group_313 | hypothetical protein |
| group_314 | hypothetical protein |
| group_315 | hypothetical protein |
| group_316 | hypothetical protein |
| group_317 | hypothetical protein |
| group_318 | hypothetical protein |
| group_319 | hypothetical protein |
| group_320 | hypothetical protein |
| group_321 | hypothetical protein |
| group_22 | hypothetical protein |
| group_395 | hypothetical protein |
| group_396 | Adaptive-response sensory-kinase SasA |
| group_397 | Response regulator ArlR |
| group_398 | hypothetical protein |
| group_399 | hypothetical protein |
| yxlF_2 | putative ABC transporter ATP-binding protein YxlF |
| lmrA | Multidrug resistance ABC transporter ATP-binding and permease protein |
| group_402 | hypothetical protein |
| group_403 | hypothetical protein |
| group_404 | hypothetical protein |
| group_405 | hypothetical protein |
| group_406 | hypothetical protein |
| group_407 | hypothetical protein |
| group_408 | hypothetical protein |
| group_409 | hypothetical protein |
| Int-Tn | Transposase from transposon Tn916 |
| group_411 | hypothetical protein |
| group_412 | hypothetical protein |
| group_413 | hypothetical protein |
| group_414 | hypothetical protein |
| tet(M) | tetracycline resistance ribosomal protection protein Tet(M) |
| group_416 | hypothetical protein |
| group_417 | hypothetical protein |
| group_418 | hypothetical protein |
| group_419 | hypothetical protein |
| group_420 | hypothetical protein |
| group_421 | hypothetical protein |
| group_422 | hypothetical protein |
| group_423 | hypothetical protein |
| group_424 | hypothetical protein |
| group_425 | hypothetical protein |
| group_426 | hypothetical protein |
| pcrA_2 | ATP-dependent DNA helicase PcrA |
| group_428 | hypothetical protein |
| pezT | Toxin PezT |
| group_430 | hypothetical protein |
| group_431 | DNA primase |
| degU | Transcriptional regulatory protein DegU |
| group_433 | hypothetical protein |
| group_434 | hypothetical protein |
| group_435 | hypothetical protein |
| bcrA_2 | Bacitracin transport ATP-binding protein BcrA |
| lagD | Lactococcin-G-processing and transport ATP-binding protein LagD |
| group_438 | hypothetical protein |
| group_439 | hypothetical protein |
| group_440 | hypothetical protein |
| group_441 | hypothetical protein |
| group_442 | hypothetical protein |
| group_443 | hypothetical protein |
| group_444 | 2-methoxy-6-polyprenyl-1,4-benzoquinol methylase, mitochondrial |
| aadK | Aminoglycoside 6-adenylyltransferase |
| apt_1 | Adenine phosphoribosyltransferase |
| group_447 | IS1380 family transposase ISSsu5 |

| Gene | Annotation |
|---|---|
| group_448 | hypothetical protein |
| group_449 | Lantibiotic macedovicin |
| group_450 | hypothetical protein |
| group_451 | hypothetical protein |
| group_452 | hypothetical protein |
| group_453 | hypothetical protein |
| glgP | Glycogen phosphorylase |

**Appendix 2 Table 4.** Genes lacking in in the ST7 isolates of this study, but present in Lineage III ST7 reference strains

| Gene | Annotation |
|---|---|
| group_173 | hypothetical protein |
| group_127 | hypothetical protein |
| group_179 | hypothetical protein |
| group_18 | hypothetical protein |
| entS | Enterobactin exporter EntS |
| msr(D) | ABC-F type ribosomal protection protein Msr(D) |
| group_182 | hypothetical protein |
| group_183 | hypothetical protein |
| dinB_2 | DNA polymerase IV |
| group_185 | hypothetical protein |
| group_186 | hypothetical protein |
| group_187 | hypothetical protein |
| group_188 | hypothetical protein |
| group_189 | hypothetical protein |
| group_190 | hypothetical protein |
| group_191 | hypothetical protein |
| group_192 | hypothetical protein |
| group_193 | hypothetical protein |
| group_194 | hypothetical protein |
| group_195 | hypothetical protein |
| group_196 | hypothetical protein |
| group_197 | hypothetical protein |
| group_198 | hypothetical protein |
| group_199 | hypothetical protein |
| group_200 | hypothetical protein |
| metK_1 | S-adenosylmethionine synthase |
| group_202 | hypothetical protein |
| group_203 | hypothetical protein |
| group_204 | hypothetical protein |
| group_205 | hypothetical protein |
| group_206 | hypothetical protein |
| group_207 | hypothetical protein |
| group_208 | hypothetical protein |
| group_209 | hypothetical protein |
| group_210 | hypothetical protein |
| group_211 | hypothetical protein |
| group_212 | hypothetical protein |
| group_213 | hypothetical protein |
| group_214 | hypothetical protein |
| group_215 | hypothetical protein |
| clpP_1 | ATP-dependent Clp protease proteolytic subunit |
| group_217 | hypothetical protein |
| group_218 | hypothetical protein |
| group_219 | hypothetical protein |
| group_220 | hypothetical protein |
| group_221 | hypothetical protein |
| group_222 | hypothetical protein |
| group_223 | hypothetical protein |
| smc_1 | Chromosome partition protein Smc |
| group_225 | hypothetical protein |
| group_226 | hypothetical protein |
| group_227 | hypothetical protein |
| group_228 | hypothetical protein |
| group_230 | hypothetical protein |
| group_231 | hypothetical protein |
| group_232 | hypothetical protein |
| group_233 | hypothetical protein |
| group_234 | hypothetical protein |

| Gene | Annotation |
|---|---|
| group_235 | hypothetical protein |
| aphA | Aminoglycoside 3'-phosphotransferase |
| satA | Streptothricin acetyltransferase A |
| group_238 | Aminoglycoside 6-adenylyltransferase |
| group_239 | hypothetical protein |
| group_241 | hypothetical protein |
| group_242 | hypothetical protein |
| group_243 | hypothetical protein |
| group_244 | hypothetical protein |
| group_245 | hypothetical protein |
| group_250 | hypothetical protein |
| group_251 | hypothetical protein |
| group_253 | hypothetical protein |
| group_254 | hypothetical protein |
| group_255 | hypothetical protein |
| group_256 | hypothetical protein |
| group_257 | hypothetical protein |
| group_258 | hypothetical protein |
| noc | Nucleoid occlusion protein |
| group_260 | hypothetical protein |
| group_261 | hypothetical protein |
| group_262 | hypothetical protein |
| group_263 | hypothetical protein |
| group_264 | hypothetical protein |
| group_265 | DNA primase |
| group_267 | Toxin PezT |
| group_268 | hypothetical protein |
| group_269 | hypothetical protein |
| group_270 | hypothetical protein |
| group_271 | hypothetical protein |
| group_272 | hypothetical protein |
| nisP | Nisin leader peptide-processing serine protease NisP |
| regX3 | Sensory transduction protein regX3 |
| creC | Sensor protein CreC |
| bcrA_1 | Bacitracin transport ATP-binding protein BcrA |
| group_277 | hypothetical protein |
| group_278 | hypothetical protein |
| nsuA | Lantibiotic nisin-U |
| nisB | Nisin biosynthesis protein NisB |
| group_281 | putative ABC transporter ATP-binding protein |
| nisC | Nisin biosynthesis protein NisC |
| nisI_1 | Nisin immunity protein |
| group_284 | hypothetical protein |
| group_289 | hypothetical protein |
| group_292 | hypothetical protein |
| group_293 | hypothetical protein |
| group_294 | hypothetical protein |
| group_295 | PTS system mannose-specific EIID component |
| sorC_2 | PTS system sorbose-specific EIIC component |
| sorB_2 | PTS system sorbose-specific EIIB component |
| group_298 | hypothetical protein |
| group_299 | hypothetical protein |
| xylB | Xylulose kinase |
| xylA | Xylose isomerase |
| nagC | N-acetylglucosamine repressor |
| group_303 | ISL3 family transposase ISSsu13 |
| group_304 | hypothetical protein |
| group_32 | IS110 family transposase ISCARN28 |
| group_71 | hypothetical protein |
| group_78 | hypothetical protein |
| gmuD_3 | 6-phospho-beta-glucosidase GmuD |
| group_15 | hypothetical protein |
| purD_2 | Phosphoribosylamine--glycine ligase |
| group_290 | hypothetical protein |
| group_3 | IS110 family transposase ISSsu7 |
| group_31 | IS110 family transposase ISCARN28 |
| group_34 | IS110 family transposase ISCARN28 |
| group_35 | IS110 family transposase ISCARN28 |
| group_39 | hypothetical protein |

| Gene | Annotation |
|---|---|
| mraY_1 | Phospho-N-acetylmuramoyl-pentapeptide-transferase |
| group_4 | IS110 family transposase ISSsu7 |
| group_43 | hypothetical protein |
| glf_1 | UDP-galactopyranose mutase |
| group_61 | hypothetical protein |
| group_65 | hypothetical protein |
| group_67 | hypothetical protein |
| group_7 | IS110 family transposase ISSsu7 |
| group_96 | IS4 family transposase ISSsu2 |

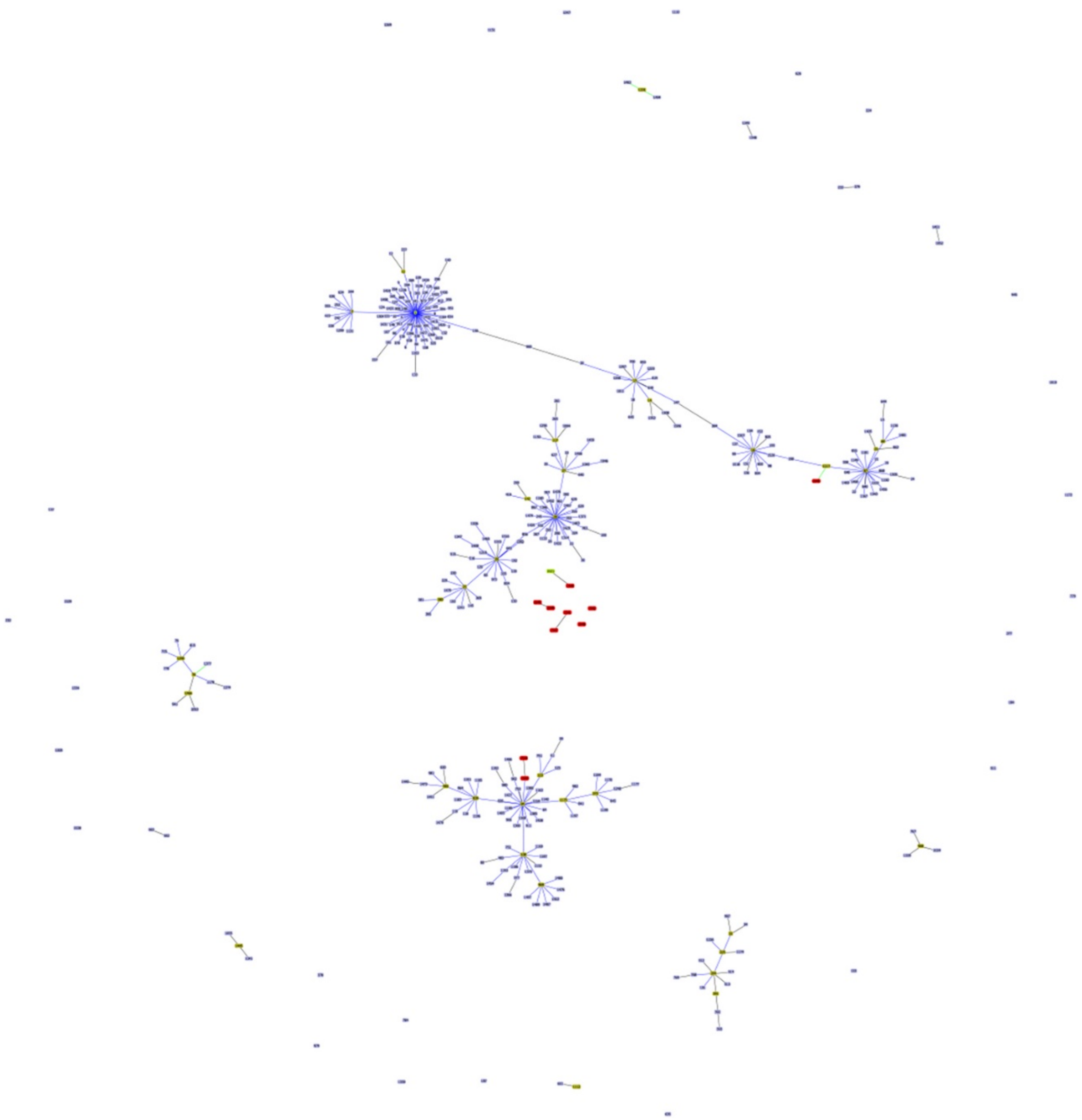

**Appendix 2 Figure 1.** e-BURST illustration of the Streptococcus suis population was used together with the complete MLST database to cluster the sequence types (STs) into major clonal complexes (CCs). The representation of groups was performed with the double-locus variants (DLVs) parameters. Primary founders (green) are positioned at the center of the cluster and subgroup founders are showed in yellow. The new STs described in our study are highlighted in red.

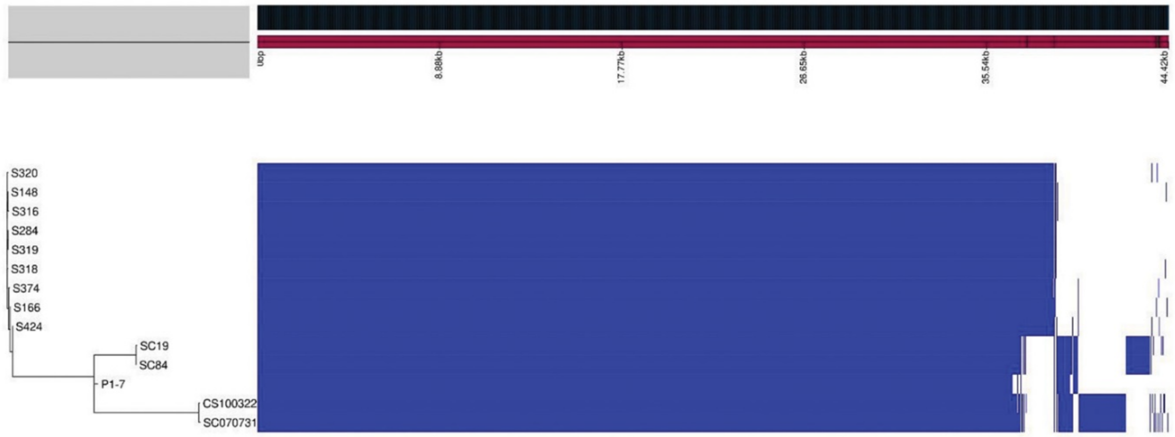


**Appendix 2 Figure 2.** Results of comparison between the genomes of our ST7 with the genomes of CS100322 and SC070731, two novel ST7 types described by Dong et al. (*7*) as belonging to lineage **III**, with SC84 and SC19, two ST7 lineage **I** strains, and with P1-7 as reference strain. The figure was generated using Phandango, an interactive viewer for bacterial population genomics (https://jameshadfield.github.io/phandango/#/) using as input the file generated by Roary (gene_precence_absence.csv and accessory_binary_genes.fa.newick). Genes are shown as light blue bricks along the top and are sorted left to right by the proportion of isolates they are observed in. Presence (blue) and absence (white) of genes are plotted considering the phylogenetic placement of each isolates.

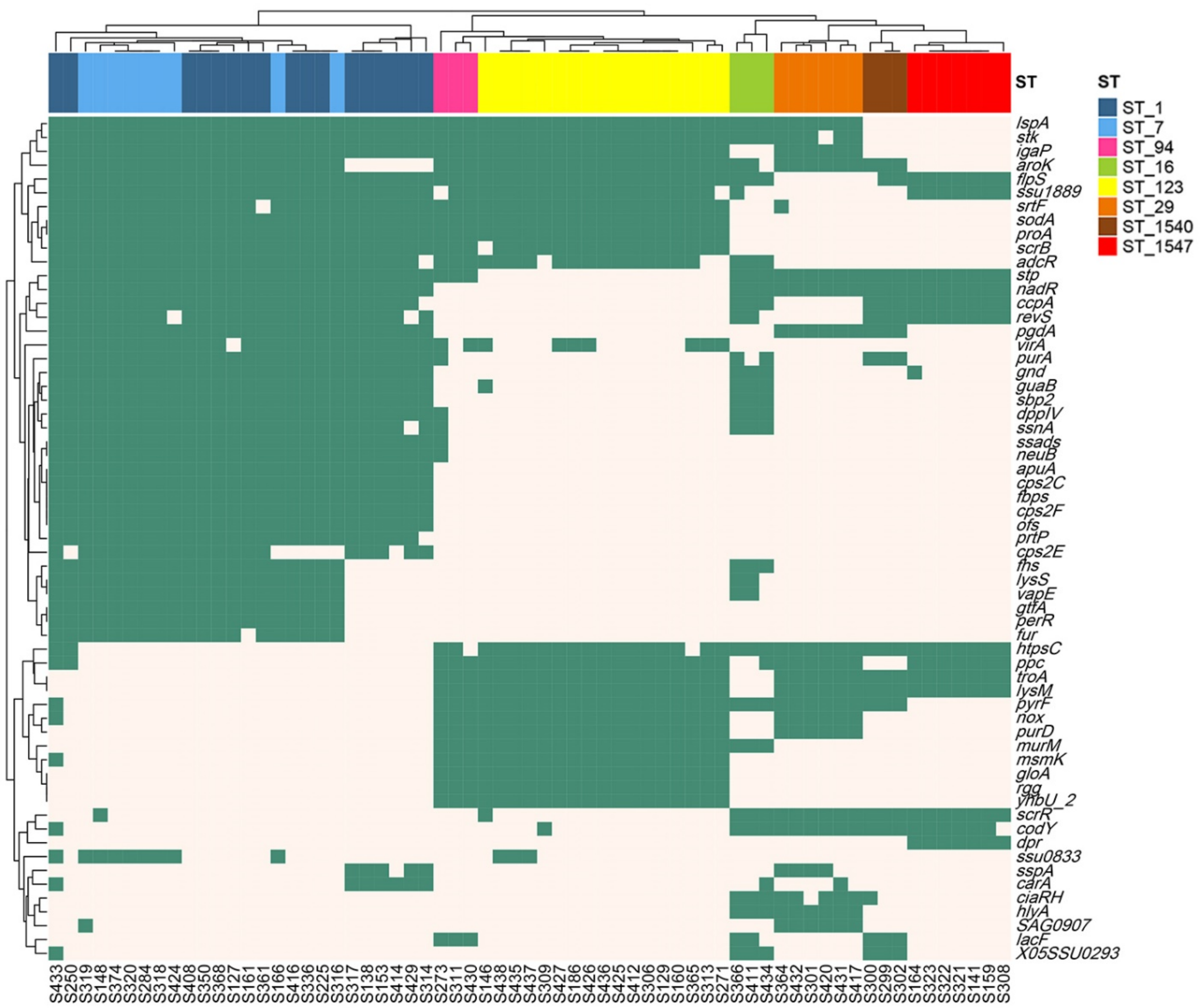
ST
ST
ST_1
ST_7
ST_94
ST_16
ST_123
ST_29
ST_1540
ST_1547
lspA
stk
igaP
aroK
flpS
ssu1889
srtF
sodA
proA
scrB
adcR
stp
nadR
ccpA
revS
pgdA
virA
purA
gnd
guaB
sbp2
dppIV
ssnA
ssads
neuB
apuA
cps2C
fbps
cps2F
ofs
prtP
cps2E
fhs
lysS
vapE
gtfA
perR
fur
htpsC
ppc
troA
lysM
pyrF
nox
purD
murM
msmK
gloA
rgg
yhbU_2
scrR
codY
dpr
ssu0833
sspA
carA
ciaRH
hlyA
SAG0907
lacF
X05SSU0293
S433 S250 S319 S148 S374 S320 S284 S318 S424 S408 S350 S368 S127 S161 S361 S166 S416 S336 S225 S316 S317 S138 S153 S414 S429 S314 S273 S311 S430 S146 S438 S435 S437 S309 S427 S186 S426 S436 S425 S412 S306 S129 S160 S365 S313 S271 S366 S411 S434 S364 S432 S301 S420 S431 S417 S300 S299 S302 S164 S323 S322 S321 S141 S159 S308
